# Supplementary material for: Stable isotope chemistry reveals plant-dominant diet among early foragers on the Andean Altiplano, 9.0–6.5 cal. ka
Source: PLoS One. 2024 Jan 24;19(1):e0296420. doi: 10.1371/journal.pone.0296420 (PMC10807835; doi:10.1371/journal.pone.0296420)
Supplement: Attachment — Submitted filename: ChenEtAl2023_PLoSONEv2_response.pdf [file pone.0296420.s004.pdf]

**Editor:** Please include the following items when submitting your revised manuscript:

- A rebuttal letter that responds to each point raised by the academic editor and reviewer(s). You should upload this letter as a separate file labeled 'Response to Reviewers'.
  **Author response:** check
- A marked-up copy of your manuscript that highlights changes made to the original version. You should upload this as a separate file labeled 'Revised Manuscript with Track Changes'.
  **Author response**: check. Note that this requirement conflicts with the journal requirement of a LaTex submission. See comment and response below.
- An unmarked version of your revised paper without tracked changes. You should upload this as a separate file labeled 'Manuscript'.
  **Author response**: check

**Editor:** The manuscript organization does not match the submission guidelines.
**Author response:** We have edited to match subission guidelines including the following

- docx format
- double spaced the text
- presented materials and methods before results
- moved author contribution to beginning of ms and edited to conform to CRediT taxonomy.
- removed funding statement from ms
- checked reference formatting
- Figures have been removed with figure captions left at the appropriate location in the text.
- Tables have been embedded in the text after the paragraph of their respective first citations.

**Editor:** You need to include more details regarding the sample composition as well as add the estimated sex and age of the individuals investigated. Additional analyses comparing sex and age groups would add further information.
**Author response:** We agree that conducting isotopic analyses by age and sex would be interesting. However, we believe those data to be related to different questions, which we will address in future work. The current paper has the specific objective of understanding adult diet among early foragers of the Altiplano. We provide the data necessary to answer this question and for other scholars to reproduce the results. We wish to maintain this problem-oriented focus, which permits the strongest possible evaluation by our reviewers.

**Editor:** The isotopic methodology used and its application in archaeology must be expanded
**Author response:** We have extended the isotopic methods per the comments of the three reviewers. Please refer to our reviewer-specific responses below.

**Editor:** More details on the Bayesian mixing models are also necessary. Since it is possible to use prior information would be important to model the human diet composition by evaluating the differential contribution of C3, C4 plants and animals feeding on such resources.
**Author response:** We use uniformed priors, and explain this in our response to Reviewer 2 below. We have also now added more information on modeling methods (see M&M), model diagnostics (see Table 4), and a posterior distribution plot (Figure 2). Finally, we have added our model code for complete transparency.

**Editor:** In general, the materials and methods are poorly described including the dataset investigated.
**Author response:** This comment seems to relate back to the earlier comments about including additional details about the human population and the mixing model. Regarding the former, as noted

above, we feel that we have provided all necessary demographic data for evaluating and reproducing the analysis as pertains to the question at hand. For the latter, see previous comment where we describe how we've expanded our presentation of the Bayesian mixing model methods.

**Editor:** The high nitrogen values found among some adults/adolescents ≥ 10‰ are interesting. In some cases, in the Andes people used animal-derived fertilizers which could increase the nitrogen values, have you evaluated such a possibility?
**Author response:** We also have noticed this interesting pattern and agree that it requires explanation. However, the current analysis is concerned with population-level patterns, and future work will address intra-population questions including age and sex based variation. The salient observation for this analysis is that all individuals in the population cluster rather tightly.

**Editor:** In the discussion, what do you mean by geographic evidence?
**Author response:** This refers to the genetic principal that a species' geographic origin in the past is indicated by region of greatest genetic diversity in the present (e.g., Out-of-Africa hypothesis supported by genetic diversity in Africa). In hindsight, this detail seems trivial. We have therefore simplified the sentence as follows: "...they are most likely associated with wild potato species, which are concentrated in the region and were likely domesticated there." We have also added two additional references to further support this statement.

**Journal requirements**
**Journal requirements:** Please ensure that your manuscript meets PLOS ONE's style requirements, including those for file naming. The PLOS ONE style templates can be found at

https://journals.plos.org/plosone/s/file?id=wjVg/PLOSOne_formatting_sample_main_body.pdf and

https://journals.plos.org/plosone/s/file?id=ba62/
PLOSOne_formatting_sample_title_authors_affiliations.pdf
**Author response:** We have attended to journal style edits. See response to previous comment to Editor above.

**Journal requirements:** We note that the grant information you provided in the 'Funding Information' and 'Financial Disclosure' sections do not match.
**Author response:** We have removed the funding statement from the manuscript per PLOS guidelines.

When you resubmit, please ensure that you provide the correct grant numbers for the awards you received for your study in the 'Funding Information' section.
**Author response:** We have added all relevant grant numbers.

**Journal requirements:** Please update your submission to use the PLOS LaTeX template. The template and more information on our requirements for LaTeX submissions can be found at http://journals.plos.org/plosone/s/latex.
**Author response:** This requirement conflicts with the Editor's instructions to provide a Word-formatted marked up copy of the manuscript (see above), which cannot be done in LaTex. We have opted to work outside of LaTex for now, but we are prepared to migrate the mansucript to LaTex format should it be accepted for publication.

**Journal requirements:** Please include your full ethics statement in the 'Methods' section of your manuscript file. In your statement, please include the full name of the IRB or ethics committee who

approved or waived your study, as well as whether or not you obtained informed written or verbal consent. If consent was waived for your study, please include this information in your statement as well.
**Author response:** we have added our ethic statement to the methods section of the manuscript.

**Journal requirements:** We note that you have referenced (Samantha M. Michell Animals of the cloud forest: isotopic variation of archaeological faunal remains from Kuelap, Peru. Unpublished Ph.D. dissertation. University of Central Florida, Orlando. (2014)) and (Watson JT, McClelland J. Distinguishing human from non-human animal bone. Unpublished manuscript of the Arizona State Museum. Tucson 2018) which have currently not yet been accepted for publication. Please remove this from your References and amend this to state in the body of your manuscript: (ie "Bewick et al. [Unpublished]") as detailed online in our guide for authors
http://journals.plos.org/plosone/s/submission-guidelines#loc-reference-style
**Author response:** We have removed Michell from citations and included their data in the supplement with in-text references to unpublished work. We replaced the Watson and McClelland reference with a published refrerence.

**Journal requirements:** We note that Figure 1 in your submission contain copyrighted images. All PLOS content is published under the Creative Commons Attribution License (CC BY 4.0), which means that the manuscript, images, and Supporting Information files will be freely available online, and any third party is permitted to access, download, copy, distribute, and use these materials in any way, even commercially, with proper attribution. For more information, see our copyright guidelines: http://journals.plos.org/plosone/s/licenses-and-copyright.

We require you to either (1) present written permission from the copyright holder to publish these figures specifically under the CC BY 4.0 license, or (2) remove the figures from your submission:

1. You may seek permission from the original copyright holder of Figure 1 to publish the content specifically under the CC BY 4.0 license.

We recommend that you contact the original copyright holder with the Content Permission Form (http://journals.plos.org/plosone/s/file?id=7c09/content-permission-form.pdf) and the following text:

"I request permission for the open-access journal PLOS ONE to publish XXX under the Creative Commons Attribution License (CCAL) CC BY 4.0 (http://creativecommons.org/licenses/by/4.0/). Please be aware that this license allows unrestricted use and distribution, even commercially, by third parties. Please reply and provide explicit written permission to publish XXX under a CC BY license and complete the attached form."

Please upload the completed Content Permission Form or other proof of granted permissions as an "Other" file with your submission.

In the figure caption of the copyrighted figure, please include the following text: "Reprinted from [ref] under a CC BY license, with permission from [name of publisher], original copyright [original copyright year]."

2. If you are unable to obtain permission from the original copyright holder to publish these figures under the CC BY 4.0 license or if the copyright holder's requirements are incompatible with the CC BY 4.0 license, please either i) remove the figure or ii) supply a replacement figure that complies with the CC BY 4.0 license. Please check copyright information on all replacement figures and update the figure caption with source information. If applicable, please specify in the figure caption text when a figure is similar but not identical to the original image and is therefore for illustrative purposes only.
**Author response:** We have opted to create our own original clip art and updated Fig 1 accordingly.

**Journal requirements:** Please include captions for your Supporting Information files at the end of your manuscript, and update any in-text citations to match accordingly. Please see our Supporting Information guidelines for more information: http://journals.plos.org/plosone/s/supporting-information.
**Author response:** We have added the captions for S1—S3 and modified the in-text reference to conform to PloS standard.

**Reviewer 1**
**Reviewer 1:** First, the authors omit basic information. Without it, it is impossible to evaluate a manuscript on stable isotopes: C/N of each sample, %C, %N and recovered collagen yield. It is also relevant to include the internal standards used in the laboratories.
**Author response:** We now present C/N, %C, and %N values with the addition of S2 Table. We have also added C/N values to Table 2.

**Reviewer 1:** Second, the terrestrial fauna values used in their model are not appropriate. Well, they correspond to animals associated with late Holocene chronologies, even associated with agropastoral contexts. Much more appropriate are the values exposed in López et al. 2017, Mondini & Panarello 2014, and Grant et al. 2018. Also, the authors do not discuss values generated for contemporary and Andean cases, as is the case exposed in Killian Galván et al. 2016.
**Author response:** We have added the data from Lopez et al 2017, Grant et al 2018, and Killian et al (see later comment below). We did not include Mondini and Panarello 2018 because that is a published manuscript that does not include the data. We have also indicated which samples are archaeological and which are modern in S2 Table. Note that we attempted to use archaeological samples when possible, but incoroporated modern samples as needed. In such cases, we adjusted for Seuss effects as indicated in S2 Table.

**Reviewer 1:** The information about the isotopic composition of the resources is poorly presented: the supplementary table does not indicate the scientific or common names of all the species and does not include the isotopic information of the archaeofauna and botanical specimens found in the archaeological site.
**Author response:** Our updated S2 Table now includes scientific names as well as additional information on sample types (archaeological or modern).

**Reviewer 1:** Lastly, the conclusion reached by the authors is disruptive but is not supported by the information they present. Half of the individuals analyzed show diets based on the consumption of terrestrial animals. This is observed with the coincidences between the estimated diets and the ellipse corresponding to that resource (figure 1).
**Author response:** Respectfully, this comment follows from a fundamental misunderstanding of isotopic process. Consumer values tend toward the average values of their foods. The fact that SMP/WMP values overlap with the ranges of mammalian values does not mean that mammals were the primary food resources. In fact, the SMP/WMP individuals overlap with the 95% ranges of both C3 plants and mammals, but all human values fall closer to the means of C3 plants, indicating that C3 plants comprised the greater proportion of the human diets. This is precisely why the Bayesian Mixing Model identifies C3 plants as comprising such a large share of the human diets. This is a common source of confusion, so we have clarified our expectations as follows in the Introdcution: "...we should expect to observe the human osteological samples from Soro Mik'aya Patjxa and Wilamaya Patjxa to exhibit dietary carbon ($\delta^{13}C_{diet}$) and nitrogen ($\delta^{15}N_{diet}$) values between those of local fauna and flora with a bias toward the means of the faunal $\delta^{13}C$ and $\delta^{15}N$ values."

**Reviewer 1:** In summary, the low values (not "enriched", please do not misuse this term), both carbon and nitrogen, could be perfectly explained by the consumption of tubers. But for this, we must first ensure that the values are primary and that the resources to infer the diet are credible.
**Author response:** The average human d15N values are not commensurate with the the mean of C3 plant d15N values. The average of the human value is elevated above the average C3 plant values, and thus "slightly enriched." This is qualitatively evident in Figure 1, quantitatively evident in the summary stats in Table 1, and confirmed by the mixing model results, which show that a 100% C3 plant diet does not offer a plausible explanation for the human data (see Table 4). We therefore do not feel we have misused the term.

**Reviewer 1:** Suggested bibliography for authors
-López M, P., Cartajena, I., Loyola, R., Núñez, L., & Carrasco, C. (2017). The use of hunting and herding spaces: Stable isotope analysis of Late Archaic and Early Formative camelids in the Tulan transect (Puna de Atacama, Chile). International Journal of Osteoarchaeology, 27(6), 1059-1069.
- Mondini, M., & Panarello, H. (2014). Isotopic evidence in Holocene camelids from the southern Puna. In Libro de Resúmenes, 12th International Conference of the International Council for ArchaeoZoology (Vol. 114).
- Grant, J., Mondini, M., & Panarello, H. O. (2018). Carbon and nitrogen isotopic ecology of Holocene camelids in the Southern Puna (Antofagasta de la Sierra, Catamarca, Argentina): Archaeological and environmental implications. Journal of Archaeological Science: Reports, 18, 637-647.
- Killian Galván, V., Martínez, J., Cherkinsky, A., Mondini, M., & Panarello, H. (2016). Stable isotope analysis on human remains from the final Early Holocene in the southern Puna of Argentina: the case of Peñas de las Trampas 1.1. Environmental Archaeology, 21(1), 1-10.
**Author response:** We have added reference to Galván et al's isotopic analysis of early Holocene individuals in the southern Puna. We have furthermore included Lopez etal, Grant et al, and Killian et al data in our baseline date (S2 Table).

**Reviewer 2**
**Reviewer 2:** The most serious issue I have with the data interpretation concerns the nitrogen trophic enrichment factor (TEF) used by the authors, which they set at 6 per mil. Within a food web, an enrichment of 15N relative to 14N in proteinaceous tissues occurs between trophic positions. This is most often cited as being about 3-5 per mil, with carnivores being 3-5 per mil higher in d15N values than their primary prey. A recent paper that includes a summary of previous collagen-to-collagen trophic enrichment factors found that a d15N TEF of 3.2 +- 1.8 was best at predicting diet (Krajcarz, Krajcarz, and Bocherens 2018). Additionally, in a presentation of the best practices for stable isotope mixing models Cheung & Szpak (2020) use a value of 4.0 +- 0.74 for the nitrogen TEF. In contrast to previous studies such as these, Chen and colleagues cite a paper by O'Connell et al. (2012) that studied d15N values in human red blood cells (not bone). From the blood data, the study then made a series of calculations to convert the blood data into collagen data, suggesting a value of about 6 per mil separated d15N from collagen and diet. This is notably very different than most other studies and from the accepted norm. Because a TEF of 6 per mil is nearly double what many scholars would consider to be an acceptable value for trophic enrichment (i.e. ~3), Chen and colleagues are essentially biasing the data towards a plant-focused diet instead of a diet that included significant herbivore meat, which indeed the zooarchaeological data from these two sites and from the broader region suggest.

The mean d15N value of the adults in the present study is 9.5 per mil while the mean d15N value of terrestrial mammals is 5.7, which means that the human-herbivore offset is 3.8 per mil. An offset of 3.8 is actually higher than the mean TEF suggested by Krajcarz et al. (2018) and within the range of the TEF suggested by Cheung & Szpak (2020). Using a more commonly accepted TEF would thus indicate that the humans from the SMP and WMP sites were primarily consuming large terrestrial

mammals for their protein source. This is a very different conclusion than the one arrived at by Cheung et al. Because the entire argument of this paper relies on the interpretation of the nitrogen isotope data, their use of this exceptionally large TEF value needs further justification, or it should be changed to a lower, more commonly accepted value. Currently, I am unconvinced that these isotopic data imply low mean consumption. In fact, to me, they look like large herbivores were the dominant protein source.
**Author response:** We have re-evaluated our data in light of this concern by examining the range of possible TEF values from 3:6 for d15N and 4:6 for d13C and incorporating the suggested error terms of +/-0.74 and +/-0.63, respectively following Cheung and Szpak 2021. We furthermore identified the most appropriate offset(s) using leave-one-out cross validation information criterion (LOOic), which shows 3 permil d15N and 4.5–6.0 permil d13C offsets to offer the best-fit models. Our updated Figure 1 graphic shows TEFs of 3 permil for d15N and 5.5 permil for d13C. Regardless of which TEF values are used, all models, which consider all possible combinations of d15N and d13C TEFs, give the same basic result that C3 plants were dominant and mammals were secondary. All results indicating a median value of 60–94% C3 plant diet and 3–33% mammal. This shows that the data are relatively insensitive to the TEF value, at least as pertains to the question at hand and scale of analysis. We have detailed these updated methods and adjusted our results accordingly. Given that this update entails a more complex modeling effort, we have also added our code for precision and transparency (S1 Code).

**Reviewer 2:** Similarly, there are some issues with the use of the MixSIAR Bayesian mixing model. The authors do not mention what TEF and error range they use for the model. I infer that it was 6, but what was the error. What would the results be if the model was rerun with a TEF of 4 +-0.74?
**Author response:** We have clarified this in our previous response. In a nutshell, we have now run the model under multiple TEFs using the recommended variance terms for both C and N.

**Reviewer 2:** Also, one of the advantages of using a Bayesian stable isotope mixing model is the ability to incorporate prior knowledge into the model. Did the authors take advantage of the zooarchaeological and paleoethnobotanical data and use the prior function within the model? Or did they use all uninformed priors? (1, 1, 1, 1) Please state.
**Author response:** We've used uniformed priors, and have updated the text to be explicit on this. The differential preservation of plant and animal evidence in the archaeological record makes it impossible to derive prior knowledge on their relative contributions to human diets. Whereas animal bone commonly preserves, the correlates of plant foods rarely preserve only when some portion of the food is accidentally burned. In fact, this preservation bias seems to be biasing our current understanding of hunter-gatherer diets. We therefore cannot use this info as an informative prior in our mixing models. We have stated this rationale in the materials and methods section as follows: "We assume uniformed priors given the lack of any prior knowledge on the relative dietary contributions of the resource classes. Although it might be tempting to draw on zooarchaeological and archaeobotanical data for informative priors, differential preservation of faunal and floral artifacts precludes this possibility."

**Reviewer 2:** Page 2, line 35: "For example, Rick [4]290 concluded that, "The settlement pattern [of the Junín region] and the faunal collections [from the site of Pachamachay] strongly support the hypothesis that vicuña, or similar camelids, were the major food source for puna [ecosystem] hunter-gatherers." – This seems like an unnecessary quote that could be put into own words and summarized. In fact, it would be more impactful to mention why they claimed that; perhaps the high abundance of camelid remains found with the faunal collections of X sites led researchers, such as Rick {4} to conclude that…
**Author response:** We maintain that quoting is important here. We want our readership to be assured that we are not creating a straw-man, and a direct quote offers the most assured way of avoiding such concerns. To be sure, we do not wish to convey any irreverence. We hold John Rick's scholarship in

the highest regard, and we do not wish to single him out. Rather we believe his understanding to be reflective of broader thinking precisely because he is such a prominent scholar in this field. We have furthermore cited our own work as evidence of the same assumption. With this in mind, we prefer to maintain the quote but would be happy to adjust as the Editor sees fit.

**Reviewer 2:** Page 2, line 56: “The biases of previous researchers who tend to be males from a culture in which hunting is a distinctly masculine pursuit could furthermore inflate the hunting signal [23].” – Here it sounds strange to be discussing the biases of “previous researchers” using the present tense. I suggest changing to past tense.
**Author response:** We have updated as follows: “ The biases of previous researchers who have generally been males from a culture in which hunting is a distinctly masculine pursuit...”

**Reviewer 2:** Page 2, line 58” “It was ostensibly for this reason that ethnography famously revealed plant foods to play a prominent role in forager economies, in contrast to earlier models that emphasized hunting.” – This is too vague. What do you mean by ethnography? Some ethnographies demonstrate extreme levels of hunting (e.g. Arctic). Need to be more specific to make the point here.
**Author response:** We have tempered this as follows: “It was ostensibly for this reason that ethnography famously revealed plant foods to play a prominent role in forager economies—Arctic economies aside—in contrast to earlier models that emphasized hunting [24–26]." We note that Richard Lee's [24] statement (emphasis added), which is the source of our claim, is as follows: "Men hunting and women gathering are widely observed in practice and deeply embedded in religion, mythology, and worldview (Biesele 1993). In energy terms, **apart from the Arctic**, women's subsistence work also tends to provide the bulk of the calories (Lee 1979)."

**Reviewer 2:** Page 6, line 109. Why mentioning quality control after presenting the results? Seems more logical to assure the readers that the data are credible before you present them.
**Author response:** We now present the quality control measures earlier.

**Reviewer 2:** Page 8, first sentence of discussion section: How did the faunal data indicate that plants comprised the major component of early forager diets?
**Author response:** Good catch. We have updated this sentence as follows: “The stable isotope, faunal, and paleoethnobotanical evidence from the sites of Soro Mik’aya Patjxa and Wilamaya Patjxa converge to indicate that $C_3$ plants, likely wild tubers, comprised the major component of early forager diets on the Andean Altiplano and that meat played a comparatively minor role.”

**Reviewer 3**
**Reviewer 3:** The Table “Human bone collagen isotopic results for Soro Mik’aya Patjxa and Wilamaya Patjxa Individuals” need to include %C, %N and C:N for each individual. It is essential to accept or reject the stable isotope value obtained. It is recommended to previously published values and other non-previously published ones. My question arise from the text where the authors mention, “As a quality control measure, we compare the resultant δ13C and δ15N values for three of the individuals—SMP9, SMP16, and WMP6—to values previously reported in radiocarbon analyses performed by The University of Arizona AMS laboratory and the KCCAMS facilty. We furthermore use C/N ratios in the radiocarbon samples to assess the extent of diagenetic alterations to bone collagen . ... We furthermore use C/N ratios in the radiocarbon samples to assess the extent of diagenetic alterations to bone collagen …”. This control measure, to my criteria, is not appropriated at all to publish stable isotope data today.
**Author response:** We now present C/N in Table 1 and %C, and %N with the addition of S2 Table. We update our text on quality control accordingly. We note that all but one individual show atomic C/N ratios within the acceptable range of 3.2–3.6.

**Reviewer 3:** Other aspect: how does the author consider/correct by breast-feeding in children individuals? Can you include it in the Table?
**Author response:** We clarify this in the M&M when we state, "Children (*n=4*) are excluded from the model to prevent breast-feeding effects from influencing the results" and in the Results when we state, "The best Bayesian mixing model for adolescent and adult individuals (*n=19*), excluding children to eliminate breast-feeding effects, furthermore indicates…". We are also careful throughout our results to indicate that our reconstruction is for **adult** diets.

**Reviewer 3:** I recommend a figure/map indicating where the human remains are located and where the resources to interpret the human diet are localized. It is basic as far as any diet reconstruction is based mostly on the resource base data and discrimination/fraction factor used. The authors need to be confident the stable isotope value on resources are based on samples recorded close to or at least in a region with similar climate and ecology. Nitrogen is sensitive to climate, as example.
**Author response:** We have intentionally restricted our samples to the central Andean highlands for the reasons the reviewer indicates, though we did extend beyond to increase our sample size and include taxa that are otherwise missing (e.g., taruca values are only reported from the northern highlands and many many C4 plant samples are from the northern lowlands, though some are from the Titicaca Basin). We have added locational information to our data table (S2 Table), which now includes lat, long, altitude and site name/country. We do not feel that a map adds additional value given that these are published data. We furthermore note that most previous publications on the topic do not include maps, and readers can consult the original data sources for more precise geographic information.

**Reviewer 3:** Please give more details in Supplementary Table 1 about the palnts and faunas used to reconstruct the human diet (loacgtions (Latitude/longitude) altitude, modern/archaeological, %C, %N, C:N,lab code…
**Author response:** We have added this information to our table (S2 Table).

**Reviewer 3:** A central variable to reconstruct isotopic diet is the discrimination fraction values used to evaluate the proportion of resources. The authors mention, "..Raw δ13C and δ15N values are converted to dietary values using published fractionation rates to facilitate comparison with isotopic values of potential food resources. δ13Ccollagen values were converted to δ13Cdiet values using a -5‰ offset to account for fractionation during bone incorporation. δ15Ncollagen values are converted to δ15Ndiet values with an offset of -6‰ to account for trophic-level fractionation." The autor needs to explain and justify this value. Other research proposes 3,5‰. Change in this value can change the author's interpretations.
**Author response:** We have addressed this also per Reviewer 2. See response to corresponding R2 comment.

**Reviewer 3:** At the same time, the 13C fraction between faunas bone collagen and human bone collagen need be explicit (all corrected by 5‰? animals and plants?). I recommend the authors use a plot with raw data isotopic data first, then a second plot with the data corrected by fraction. I recommend the author discuss the fraction values used and why they reject others in more detail. That is, the authors must justify the validity of the resources used and the fractionation values used to reconstruct human diets. Bernal et al. (Current Anthropology) show how strongly our interpretations of human diet reconstruction depend on these values.
**Author response:** All human-bone values are adjusted for trophic fractionation in order to reflect dietary values. This is independent of plant vs animal foods. We do not correct plant or animals for trophic fractionation since we are not analyzing their diets. However, we do include fractionation

corrections for non-trophic processes, and these are all specified in the M&M. Finally, per Reviewer 2, we have conducted the mixing model under a wide range of plausible trophic fractionation scenarios, and obtain the same basic results regardless (see Table 4).

**Reviewer 3:** The author indicates they used MixSiar to reconstruct the human diet with Bayesian statistics. Why not FRUIT? In the Supplementary material, I recommend details of the Mixiar (or Fruit) analysis.
**Author response:** In the M&M, we now present the following explanation for our choice of MixSIAR: "Although other packages for mixing modeling are available, we use MixSIAR because of its currency, documentation, integration with R statistical computing language, and accessibility with open-source Linux operating systems (Cheung and Szpak 2022, JAMT)." We have furthermore added more details for our mixing model analysis including additional info on priors (uninformed), model diagnostics (see Table 4), control sample data, and graphical output (Figure 2). Many of these additions follow from previous comments.

**Reviewer 3:** If the tuber should be the plant consumed, are the authors running Bayesian models with tubers or all C3 plants?
**Author response:** We use all C3 plants, which includes tubers. Because there is no theoretical basis for assuming that tubers should behave differently than other C3 plants, we do not subdivide our control samples. For what its worth, we had the same intuition and thus compared Solanum samples to all others. We found—not surprisingly—that Solanum values were statistically indistinguishable from the broader population of C3 plants. Again, well-established isotope theory anticipated this, and we therefore see no reason to further sub-divide our control sample for the purpose of this paper.

**Other changes**

- Since our submission, we acquired isotopic data for another individual, which increases our sample size by 1. We have included this individual (WMP 8) and updated all analyses accordingly. This has no affect on the analytical outcome, but we feel it is important to include all individuals for whom we have data. This individual was analyzed in yet a 3rd isotope lab with commensurate resutls, which should give even greater confidence in data quality.
- Indicated radiocarbon calibration curve as cited in Table 2 (2020 Southern Hemisphere Calibration Curve [47]).
- *We have removed "human bone" from the title to accommodate public sensitivities around human remains and recognizing that the term adds little to the title. The updated title is, "Stable isotope chemistry reveals plant-dominant diet among early foragers on the Andean Altiplano, 9.0–6.5 cal. ka"*
