# Supplementary material for: Stable isotope chemistry reveals plant-dominant diet among early foragers on the Andean Altiplano, 9.0–6.5 cal. ka
Source: PLoS One. 2024 Jan 24;19(1):e0296420. doi: 10.1371/journal.pone.0296420 (PMC10807835; doi:10.1371/journal.pone.0296420)
Supplement: Attachment — Submitted filename: ChenEtAl2023_PLoSONEv3_response.pdf [file pone.0296420.s005.pdf]

Editor: "...the last changes requested from reviewer 4 are truly minor."
Reviewer 4: "In Tables 1 and 2, should only have one decimal place for the C and N isotope values. Same in lines 219-220. In Table 4, the N isotope values should have one decimal place."
Author response: We have changed the significant figures accordingly.

Editor: "I want to share with the authors my opinion regarding the model used. Despite the authors having used various criteria to select the best model, the narrative that hunting was not common in the Archaic would be inconsistent with another viable model: d13C TEF of 5 and a d15N TEF of 4 (per Cheung and Szpak 2020) which would result in much lower plant contribution to the diet (75%)."
Author response: We thank the editor for this note, and we acknowledge that some models indicated plant contributions as low as 75%, and even as low as 60% (Table 4: model 1). Nonetheless, all models show that plant foods were the majority food. To be sure, we are not claiming that hunting was uncommon. To the contrary, all models indicate that meat was a part of the diet and thus was common. These data make clear that plant foraging was more common than hunting to daily subsistence practices.
